# Experiences and challenges of Prostitute Women in Iran: A phenomenological qualitative study

**DOI:** 10.1016/j.heliyon.2020.e05649

**Published:** 2020-12-03

**Authors:** Javad Yoosefi lebni, Seyed Fahim Irandoost, Arash Ziapour, Mohammad Ali Mohammadi Gharehghani, Farbod Ebadi Fard Azar, Goli Soofizad, Bahar Khosravi, Mahnaz Solhi

**Affiliations:** a Health Promotion Research Center, Iran University of Medical Sciences, Tehran, Iran; b Department of Public Health, School of Health, Urmia University of Medical Sciences, Urmia, Iran; c Health Education and Health Promotion, Health Institute, Kermanshah University of Medical Sciences, Kermanshah, Iran; d Social Welfare Management Research Center, University of Social Welfare and Rehabilitation Sciences, Tehran, Iran; e School of Public Health and Safety, Shahid Beheshti University of Medical Sciences, Tehran, Iran; f Students Research Committee, Kermanshah University of Medical Sciences, Kermanshah, Iran

**Keywords:** Psychology, Cultural sociology, Prostitute women, Experiences, Challenges, Qualitative study, Iran

## Abstract

**Background:**

Prostitutes in Iran are faced with many challenges and problems that pose risks to their health.

**Objective:**

The present study is an attempt to identify and narrate the challenges and experiences of Iranian prostitutes based on a qualitative approach.

**Methods:**

This qualitative study was conducted with a phenomenological approach in (2018) in Tehran, Iran. The data were collected through semi-structured interviews with 22 prostitutes who were selected using a snowball sampling method and analyzed with Colaizzi's method. In order to examine the quality of findings, Guba and Lincoln's measures were used.

**Results:**

Data analysis results were classified into five main categories and 14 subcategories. The main issues are: The experience of violence, Heath risk, social ostracism, objectifying, and lack of social and legal supporting structures.

**Conclusion:**

Prostitutes in Iran experience numerous problems at personal and social levels. By providing social, economic, and legal supports for them such as social services (e.g. educations on how to use contraceptives, how to have safe sexual intercourse, and free counseling services for mental support), we can improve their health and welfare.

## 1 Introduction

Prostitution refers to the sale of people, especially women and children, for other's sexual pleasure. Prostitution is the invasion and exploitation of fundamental human rights and dignity [1]. Prostitution is an existing phenomenon since ancient times, and today also it exists almost all over the world, which in some places accepted and in some other considered as a sin [2]. In some countries, there is a functional look at prostitution, and in others it is considered a crime and prostitutes are punished. Considering prostitution, either a crime or a function, it is undeniable that there are many problems for the person and the community involved [3, 4].

At the individual level, prostituted women have tough lives and experience numerous challenges. Women involved in prostitution are at high risk of developing HIV [5, 6]. The ever-increasing of HIV infection among women worldwide has been posed as a global challenge due to the massive impact on health organizations’ reproductive health [7]. The life probability of women with HIV is 13 times higher than that of all women of reproductive age in low and middle - income countries [8]. It is estimated that approximately one eighth (11.8%) of prostituted women in developing countries are infected with HIV [9], while according to reports from 38 countries, on average, only 58% of these women have access to health services for the prevention of AIDS and STDs [10]. In Cambodia, the rate of HIV prevalence in prostituted women is between 13.9% and 17.4% [11, 12].

Farley M et al., 2004 and Ma M et al., 2018 mentioned some of the life experiences of prostituted women: all prostituted women commonly experience sexual violence and physical assault 70 to 95 percent of prostituted women in their study experienced physical violence. Health problems such as fatigue, viral diseases, backache, depression, headaches, etc., are also very prominent in prostituted women, and they may even be killed [13, 14]. Evans E et al., 2019 studied prostitutes in El Salvador and highlighted problems like emotional and economic violence, lack of access to legal, health social services, and loss of income, job, housing, and educational opportunities [15]. Duff P et al., 2017 showed in a study in Uganda that prostitutes had several health needs in pregnancy and HIV fields in particular and Unplanned pregnancy was one of the main issues [16].

Some studies reported a significant relationship between drug abuse and deviant behavior, in which 60% of women using cocaine had a prostitution record [17]. In another study, 66 percent of prostitutes used drugs [18]. McTavish J 2010 mentions the prostituted women's rights, which should be provided to these women as "human beings", in the following areas: treating them as human beings, not treating them unjustly, legal protection of them, not abusing them, the right to have health and social services [1]. Meanwhile in countries where prostitution is illegal, especially in Muslim communities, prostituted women are deprived of social rights and are considered social parasites [19].

Prostitution in Iran began in the Qajar period with the creation of a brothel named Qala-e-Shahr-e-Naw in Tehran. Then, during the Pahlavi era, it became more intense, and special rules were passed to regulate the prostitutes and their clients' behavior so that more than a thousand prostitutes worked there. On the Islamic Revolution outbreak in 1978, prostitution was considered a crime, and the brothel was destroyed; most of the women who worked there were imprisoned and some executed [20]. In Iran, because prostitution is illegal, there are no accurate statistics on the number of prostitutes, but new researches show that prostitution has increased again in recent years [21, 22, 23] and it is estimated that there are about 228,700 prostitutes in Iran [24].

In Iran, prostituted women get social labels, and the country's social and cultural environment exposes the lives of women involved in prostitution to abuse and violence [25, 26]. A study in Iran in 2017 showed that street prostitutes usually do not use condoms in sexual intercourse due to the desire of customers and lack of bargaining power, as well as old age and low awareness of sexually transmitted diseases that cause them to face many problems [27]. Other research results in 2018 in Iran also indicated that prostitutes have little access to health services such as HIV testing and counseling [28].

Most studies on prostitution in Iran have been done quantitatively and experimentally, and relatively few studies with a qualitative approach have tried to identify the problems and experiences of prostitutes from their perspective. Also, prostitution, apart from the negative consequences it has for the women involved, is a challenge for health authorities and a threat to social and religious values [29, 30]. Since prostitutes are among the vulnerable social groups and along with the risks to their health, they also act as a risk to others’ health, findings the problems and challenges that they experience can be helpful for future health interventions. Therefore, it is essential to conduct qualitative studies on this topic, and the present one is an attempt to identify experiences and challenges in the face of prostitutes in Tehran.

## 2 Materials and methods

### 2.1 Design

The present study was carried out with a qualitative method through a phenomenology approach. The purpose of using the qualitative method is to understand the depth, complexity, details, and context of the phenomena studied through the researcher's active engagement in the course of the research. Phenomenology is the science of studying, describing, and interpreting the various phenomena of life. In phenomenology research, experiences, perceptions, and feelings of individuals are studied [31].

### 2.2 Setting and participants

This study's research population was prostituted women of Tehran in 2018 who were chosen based on both inclusion criteria of the research: to have a prostitution experience, willingness to participate and ability to respond and exclusion criteria: unwillingness to participate and to leave the interview. The sampling method was purposeful with a snowball strategy and, to gain maximum information, a wide variety of samples were considered, and after interviewing the first sample with a snowball pattern, she was asked to introduce a person with prostitution experience or a person engaged in prostitution. Interviews were conducted in public spaces, parks, and places to the participants' convenience, and coordination was done with the respondent to select these environments, and if she was not satisfied, another place was selected for the interview based on her opinion. Samples were selected from around the Tehran city by snowball method, but most of them belonged to Tehran's central, southern, and suburbs.

### 2.3 Data collection

The data collection process took about five months, from April to September 2018, using guide questions and semi-structured interviews and applying the individual interviewing technique. Authors 3 and 7 conducted the interviews. Author No. 3 is a Ph.D. student and has experience in conducting qualitative research and interviews. Author No. 7 is also a Master of Women Studies and a researcher in the field of gynecological injuries, which has conducted several qualitative studies on women in Iran.

This study was sponsored by the Deputy of Research and Technology at Kermanshah University of Medical Sciences (KUMS) with ethical code (IR.KUMS.REC. 1398.633). For ethical considerations and in the snowball sampling method employed by the research, to protect the participants' privacy in the interview, the first participant was asked to introduce a prostitute, make the necessary arrangements, and ask her permission. After this coordination, the research team contacted her and coordinated the appropriate time based on her opinion. At the visit time, all issues related to the research ethics were explained to her, and she was informed of the full participation authority. The researchers explained that no names or addresses of the participants were mentioned at the publication of the findings. Also, the file of recorded interviews should not be given to any legal body. Also, the interviews were conducted in a quiet place and without any other person except the researcher and the participant. Then, she signed the consent form, and with her permission to record all the conversations on tape, the interview was carried out. Since the interviews were conducted with the participants' prior coordination and consent, all were completed, and none were repeated.

The interviews would be started with a few demographical questions followed by questions like “what happened after you became a prostitute and what were the problems? How have you been treated by your sex partners and the society? Have you ever been subject to violence? Explain please. What were the health risks after becoming a prostitute? How was your feelings about yourself and the sex partner? And “what were the supports you received from civil and state bodies? Regarding the socio-economic situation, the respondents were asked which class they considered to belong to, and the results are shown in the descriptive table. During the interview, field notes were used, and participants' postures such as body language, pause, and silence, and the effects of fury on their faces were recorded.

The theoretical saturation criterion determined the number of samples; the data collection was stopped when the answers were repetitive and new data were not obtained from the interviews. Accordingly, an interview was conducted with 22 prostituted women who were in the city of Tehran. On average, each interview lasted 45–60 min. Also, the minimum interview lasted 35 min, and the maximum was 70 min. None of the participants refused to participate in the research after learning the research process's goals and necessity and how they were reported.

### 2.4 Statistical analysis

The coding was done by the fourth and fifth authors, who are university professors and health researchers. Data analysis was carried out simultaneously with data collection. Thus, after each interview, the researcher and her colleague listened to the interview content twice and transcribed the conversations on the paper. Then, using MAXQDA-12 software and the 7-step Colaizzi technique [31, 32], data analysis was performed: carefully read all the descriptions and essential findings of the participants, extracting crucial phrases and sentences related to the phenomenon, conceptualizing the extracted meaningful sentences, regularly making participants' descriptions and common concepts in specific categories, turning all inferred ideas into comprehensive and complete descriptions, turning complete descriptions of the phenomenon into an accurate, concise, and real description and referring to the participants to clarify the ideas obtained and validate them. In other words, the text of the interviews was first to read carefully, and its essential phrases were identified and recorded as code. The codes' initial analysis was written separately, and the codes with conceptual similarity were placed in a subcategory, and a name was assigned to it. By merging subcategories based on familiar concepts, main categories were created. Finally, a general and comprehensive description of the phenomenon is formulated and presented in the form of a clear statement.

### 2.5 Trustworthiness

Guba and Lincoln's trustworthiness criteria were met to assess the quality of the results [33, 34, 35]. In this regard, for reliability, the researchers devoted a great deal of time to collect data and considered triangulating (using several researchers for data collection), taking notes, and continuous observation. For reliability, a quality research specialist supervised the process, and the data was shared with several participants simultaneous with data collection and analysis of data to verify the researcher's findings by their experiences. In order to increase the validity, researchers attempted to put aside personal attitudes and record all aspects and observations, and dismiss their presumptions as far as possible in the process of data collection and analysis. It was attempted to select individuals of different socioeconomic levels, educational status, marital status, and ages and express specific reasons and meanings for each part of the community's describtion and identified attributes to make findings generalizable.

## 3 Results

The present study was conducted with the participation of 22 prostituted women in Tehran. The demographic information of the participants in the study is presented in Table 1.

**Table 1:** Demographic information of participants.

Characteristic	Frequency
Age	
Mean	28
Minimum	19
Maximum	42
Marital status	
Single	4
Married	6
Divorced/widowed	12
Education	
No high school diploma	8
Above diploma	6
No high school diploma	8
Economic status	
Low	11
Average	7
High	4
Record of prostitution	
Mean	7
Minimum	1
Maximum	14
Number of relationships a week	
Mean	4
Maximum	11
Minimum	2
Record of abortion	
Yes	18
No	4
Record of using drugs	
Yes	19
No	3

After analyzing the data, five main categories and 14 sub-categories were identified (Table 2), which is examined separately in the following.

**Table 2:** Categories and subcategories of prostituted women experiences.

Categories	Subcategories
Experience of violence	Physical violence
	Verbal and psychological violence
	Economic violence
	Sexual violence
Health risk	Suicide
	Addiction
	Sexual Diseases
	Unwanted pregnancy and unsafe abortion
Social ostracism	Social threat
	Disorder in social and personal relationships
Objectifying	Women as being commodities
	Losing feelings and emotions
Lack of social and legal supporting structures	Lack of social-legal support
	Lack of support by health-medical institutions

### 3.1 Experience of violence

Prostituted women are subject to various violence because of their economic and social conditions, which can cause much damage to them.

#### 3.1.1 Physical violence

One of the most common violence against prostituted women is physical violence. This violence occurs for various reasons; part of it is because of low status and the kind of community's view of prostituted women, and another part because prostituted women's customers know that they have no backing and support; therefore they commit physical violence against them.

*"Sometimes it happened that after sexual relationship a customer gave me a beating so that my head and face were bleeding" (27 year-married).*

*"Some days that I stand on the streets some of the cars[drivers] that knew me deliberately drive over me to hit me, one and or two times they hit me and escaped" (25 year-single).*

*"When people realize that we are prostitutes, they behave with us as they please, sometimes they give us beating" (35 year - divorced)*

#### 3.1.2 Verbal and psychological violence

Another aspect of violence against prostituted women is related to verbal and psychological violence and harm. In this regard, customers of prostituted women, their associates and society's people by using malicious words and vulgarity and inappropriate vocabulary, while having verbal violence, cause a lot of psychological harm to prostituted women.

*"Sometimes, while having sex, our customers use the worst insults and call us with the worst words (33 year- divorced)*

*"In addition to our customers, neighbors or those in the neighborhood who know we are doing this, insult us in the worst possible way all the time, they talk in a way as if we are not human beings" (40 year - widowed)*

#### 3.1.3 Economic violence

Customers’ infidelity to pay agreed on money, not paying money for prophylactic devices by customers, robbery, and stealing money from the women by customers are examples of economic violence that affects prostituted women.

*"I often agree with a customer on a certain price, but after the relationship, he dodges it and pays so much less, sometimes they do not give any money at all*" *(40 year - widowed)*

*"Sometimes, it happens we go to the house or villa with the customer's invitation, but after a couple of days, they give no money, and even it comes about that they take our money" (41 year-divorced)*

#### 3.1.4 Sexual violence

Prostituted women also face up much violence in the field of sex itself; forced sex, involuntary unusual sexual relationship, group sex by force, compulsion not to use protective and prophylactic devices, are some of this kind of violence.

*"Sometimes I came to agree with one, but when I went to the place, they forced me to have sex with several people" (35 year - divorced)*

*"Sometimes it happened that I was reluctant to have a sexual relationship, but I had to make it” (25 year-single)*

*"Some of your customers behave in such a way that we hurt and that we cannot have sex for a few days or even a few weeks" (28 year- divorced)*

### 3.2 Health risk

Due to the conditions they face, prostituted women have messy and miserable lives and are exposed to severe social and physical harm. Among the challenges of this stratum are suicide, addiction, sexually transmitted diseases, unwanted pregnancy, and unsafe abortion.

#### 3.2.1 Suicide

Prostituted women find no way but committing suicide because of their conditions and inability to change it in many cases, and they attempt to eliminate their selves.

*"Since I got into this job, my life was over. I wish death most of the days. Sometimes I think of suicide" (33 year-divorced)*

*"Sometimes my customers annoy my soul so that I'd like to die. So far, I have attempted one or two times but my friend hindered me and saved me but I wish I had died" (35 year-widowed)*

#### 3.2.2 Addiction

Addiction can be one of the main reasons for turning to prostitution and one of its main consequences, because many prostituted women have become addicted to drugs after turning to prostitution, and in most cases, this addiction is imposed on women by their customers.

*"Sometimes our customers force us or pay more money to drink wine or use opium with them in order that we can give them more pleasure" (41 year-divorced)*

*"We have to take meth crystal or other drugs to be able to have more sex. Some of us begin to use drugs to get carefree of this life and to suffer less" (38 year-widowed)*

#### 3.2.3 Sexual diseases

In some cases, prostituted women are coerced to undertake an unsafe sexual relationship leading to the transmission of sexual diseases such as AIDS and other sexually transmitted diseases.

*"Before entering the room, they are kind and everything goes as planned, but as soon as they enter the room, they show the other side of themselves and have strange requests. They often force us to have sex without a condom. Because of this, most of us take sexually transmitted diseases" (29 year-single)*

*"Once I went to a villa, I had to have an unsafe relationship with several men I was familiar with them, I knew they might have a disease but I had no choice. If I did not do it, they might kill me, so I did it. After a while, I took a blood test. I realized I was HIV positive" (28 year-divorced)*

#### 3.2.4 Unwanted pregnancy and unsafe abortion

Because prostituted women sometimes have to do unwanted and unsafe sex. In some cases they become pregnant and often abort the baby or, if they give birth, they give it to Iran's Welfare Organization centers, etc. or even they sell it. The abortions are carried out without considering hygienic measures and are dangerous and cause serious harm to women.

*"I did not know that I was pregnant; on a day when I had pain, I went to a doctor and the doctor recognized that I was pregnant; I did not know what to do; I loved to keep it; but I knew that would commit the immense oppression against it by holding it, so I aborted it illegally" (33 year-widowed)*

*"By far I have aborted twice, in one case I was near to die; I did both abortions in my friend's house" (38 year-widowed)*

### 3.3 Social ostracism

Due to their particular circumstances, prostituted women are isolated and not accepted in society and are ostracized by the family and associates.

#### 3.3.1 Social threat

One of the social ostracism aspects of prostituted women is a social threat against them. In this process, people hurt prostituted women and think they have the right to do any harm to them. They have to change their place of living because of their lack of support.

*"God forbid that some guy knows we are prostitutes, he allows himself to make us suffer any misfortune which he likes because he thinks we spoil the society. Sometimes I have to change my house every few months, and I incur a significant loss because of the neighbors' behavior" (23 year-single)*

#### 3.3.2 Disorder in social and personal relationships

Prostituted women are boycotted by their families and their associates, and their interactions are mere with customers, and they live in a very isolated and lonely situation.

*"After my family knew that I got into this job they never wanted to see me, I lost all my old friends, I'm very alone" (30 year-divorced)*

*"Since I got into this job, I have no friends. There is nobody I can talk with a little. I only have relationships with my customers who look at us as an animal as soon as they are done" (33 year-divorced)*

### 3.4 Objectifying

Objectifying is a two-way process. On the one hand, men see prostituted women as objects and commodities merely to satisfy their sexual needs. On the other hand, women, because of the variety of relationships without satisfaction and interest, regard themselves as lacking in feeling and affection, and only one object.

#### 3.4.1 Women as being commodities

Most men who refer to women prostitutes are only for the satisfaction of their sexual needs, rather than their spiritual needs and their relationship, only for a brief period, and the promise of men to prostitutes is just as a commodity for sexual satisfaction.

*"Sometimes I feel like a pizza in the restaurants that people buy and eat with eagerness, but when they get full, they throw the last piece into a trash can, and as if no one were all eager for it a couple of minutes ago. Men want us only to satisfy their instant needs" (27 year-divorced)*

#### 3.4.2 Losing feelings and emotions

At the same time as men perceive prostituted women as sexual commodities, women themselves also find this sense, and since the relationship between them and men is cut short, without any feeling and interest, they are discarded and separated from society; after a while, they come to the conclusion that they lack feelings, affection, enthusiasm, and love.

*"I have no feeling at all for a long time, I became like a stone. I feel that there is nothing in the name of interest, affection, and love inside me" (30 year-widowed)*

### 3.5 Lack of social and legal supporting structures

Another challenge facing prostitutes in Iran is the lack of supportive social and legal structures that make them less likely to receive social support, health care, and services.

#### 3.5.1 Lack of social-legal support

Prostituted women do not have security and are always harassed by others, and because there is no institution for lawful support of them, they live a hard life, and when they lodge others they are condemned in advance.

*"Some of our customers enter our house by force and bother us and we cannot do anything. If we go sue them we ourselves are more offended, we have to stay silent" (25 year-single)*

*"All people oppress us and we cannot turn to anyone for support. If we go to anyone for complaint when they know that we are a prostitute, they give the defendant the right" (28 year-divorced)*

#### 3.5.2 Lack of support by health-medical institutions

In addition to lack of legal and social support, in the field of health and access to services, prostituted women face challenges and, if they have a disease or demand for abortion, they confront the adverse reaction of the relevant institutions.

*"We have to have many troubles for abortion. No one and no organization supports us. Thus we have to abort in the worst circumstances" (33 year-divorced)*

## 4 Discussion

This study was an attempt to identify the experiences and challenges of prostitutes in Tehran. The present study showed that women's prostitution experiences are multidimensional and diverse and this forms a challenging life for them.

The first and most frequent experience in prostitution is the experience of violence. Physical violence and bodily harm to women, verbal harm, and violence, not paying the agreed money, and even theft of women's money, along with forced sex, involuntary unusual sexual relationship, group sex by force, compulsion not to use protective and prophylactic devices are some aspects of the violence which prostituted women in this study mentioned. Various studies have shown that violence against prostituted women is a common phenomenon [25, 26, 36, 37, 38, 39, 40, 41, 42]. Farley M et al., 2004 and Scorgie F et al., 2013 showed that violence against prostituted women is a norm and sexual and physical harassment, verbal humiliation, and torturing are forms of this violence [14, 43]. Sadati A et al., 2017 showed that prostitutes of Shiraz in Iran experience all kinds of physical, psychological, sexual, deception, and theft violence [27]. Sarode S 2002also found that 80% of prostituted women experienced the harms caused by violence during sexual relationships [44]. The unwillingness of sexual partners to use devices for harm reduction, lack of customer commitment to pay pledged money, and a large number of sex partners simultaneously (sex with a group of men) have been shown in various research [39, 45, 46, 47, 48]. Prostituted women are subject to various violence because of their economic and social conditions, causing a lot of harm to them. This massive violence against women imposes severe physical and psychological harm on women and it highlights the need to counteract this situation and protect them.

Harm to prostituted women has another aspect. The study results showed that suicide attempts, addiction, infection of sexually transmitted diseases, especially HIV, and unwanted pregnancy and unsafe abortion are some prostituted women's experiences in their messy lives. In various studies, the prevalence of HIV and other viral infections in prostituted women is mentioned [37, 40, 49, 50]. In Cambodia, the HIV prevalence rate in prostituted women is between 13.9% and 17.4% [11, 12]. Most of the prostituted women in this research stated that they use drugs and alcohol to give more pleasure to men and have the ability to have multiple relationships with men and in some cases voluntarily, which is also shown in other research [18, 40, 45, 46, 47, 51, 52, 53]. In the study of Mohammadi Gharehghani MA et al., 2020 in Iran, the results showed that sexual partners' desire to have more pleasure caused their reluctance to use condoms [54]. Qayyum S et al., 2013 also referred to the issue of unwanted pregnancy and abortion among prostituted women [40]. Regarding the prevalence rate and condition of diseases transmitted sexually, in particular HIV, addiction and unwanted pregnancies of prostituted women, there is a need for control and planning to reduce sexually risky behaviors. In this regard, in addition to confronting the phenomenon of prostitution, the use of preventive devices should be urged and trained, and at least sexually risky behaviors should be taken with preventive devices to prevent sexual illness and pregnancy.

Social desertion was another experience and challenges mentioned by the participants. This finding is consistent with previous studies in this field. Notably prostitutes face several social challenges and personal problems that result in their social isolation and more vulnerability in return. Qayyum S et al., 2013, showed that prostituted women are ostracized from their families and relatives, and the society looks at them very scornfully, and the only thing that their customers want them is sex [40]. Other research shows a look of reproach at prostituted women in the society and denouncing their work, in which prostituted women are ostracized and marginalized [37, 38].

Another exciting and new finding was the feeling objectified and empty of emotions in the prostitutes. These aspects have received less attention in previous studies. Given that most clients only sought after fulfilling their sexual needs and there was no emotion in their relationships with the prostitutes, many of these women felt that they were objectified. Sarode S 2015, showed that prostituted women have only a sexual and economic relationship with customers, and there is no love and affection [44]. The sense of being objectified and no emotion and feeling made the prostitutes develop negative attitudes about themselves. This sense may result in several mental problems and even affect their social relationships.

The results showed that the lack of supportive social and legal structures in society was another major challenge and a notable finding of this study. This lack of support is more pronounced when they are bullied, and all their individual and social rights are violated, and they are potentially considered criminal when they go to organizations and complain. Also, they are often not accepted when they get ill and need health services and go to health centers; for example, they do abortions that are done without considering health standards and are very dangerous. Ramaiah S 2006 and Weitzer R 2009 pointed out that prostituted women access to welfare, health care services, and the right to have healthy reproduction is compromised [37, 38]. In other studies, women have pointed out that there is no proper help and support for women involved in prostitution [3, 15, 55, 56]. In the study of Asadi-Aliabadi M et al., 2018 in Iran, the results showed that prostitutes are less likely to receive health services, and they are less inclined to perform medical tests and receive health care due to the restrictions and pressures on them [57]. Indeed, prostitution is an illegal and unethical business in Iran, and these women receive no legal or even health supports, which is a great danger to their health, and they are among the weakest and most vulnerable groups in society.

## 5 Conclusion

The results show that prostituted women at various personal, interpersonal, and social levels are subject to many harms such as the experience of violence, heath risk, social ostracism, objectifying, and lack of social and legal supporting structures. Actions should, in the first place, include creating the conditions for reducing this phenomenon, as this is not legally, culturally, and socially acceptable. However, steps can be taken to reduce the effects and challenges associated with this phenomenon. Due to the socio-cultural conditions and the illegality of prostitution, which leads to a double violation of this group of women's fundamental rights, priority measures should be taken to establish a structure to protect women against oppression and violence legally. Due to the strong religious and legal sensitivities that exist against the prostitution of married women, economic and livelihood assistance to this group of women should be a priority to not endanger their lives. Public education, particularly men, not to use violence against this group of women and emphasize that most of these women engage in prostitution due to fundamental livelihood problems can reduce public opinion's stigma and criminality against prostitutes. To improve the health condition of prostitutes, we need to be more active at all levels, which entails more cooperative attitudes in public and civil bodies. Success in this quest needs changes in the attitudes of decision-makers and NGOs, new laws, provision of social supports (e.g. educations about contraceptives and safe intercourse), and establishment of free counseling centers to empower these women with an emphasis on sexual, mental, and spiritual aspects following by economic empowerment. Future studies can focus on women's experiences and challenges in other Iran cities and show cultural and geographical differences and similarities regarding the issue. These studies can also accurately examine why married women turn to prostitution or why violence against prostitutes generally by examining the socio-cultural determinants of prostitution and general approach towards it.

## 6 Limitations and advantages

One of the study's main advantages was that the problems and experiences of prostitutes in Iran were examined qualitatively and comprehensively. Thus The results, uncover the hidden aspects of the problem and introduce effective solutions to policy makers. Another advantage of the study was the diversity of participants so that they were from different groups with different demographical specifications. This diversity helped finding in richer concepts. However, the study was not free of limitations; securing an ethical code from the university was not easy, given the issues' sensitivity. The authors tried hard to convince the university officials by elaborating on the objectives and necessity of the study. Another limitation was the fear and lack of trust in the participants. To solve this, the authors tried to discuss the study's objectives and necessity with the subjects and employed female interviewers familiar with qualitative studies. Also, the qualitative nature of the study, small sample group, and sampling method lower the generalizability of the findings.

## Declarations

### Author contribution statement

J. Yoosefi Lebni, M. Solhi: Conceived and designed the experiments; Wrote the paper.

S. F. Irandoost: Conceived and designed the experiments; Analyzed and interpreted the data; Wrote the paper.

A. Ziapour, B. Khosravi: Performed the experiments; Contributed reagents, materials, analysis tools, or data.

M. A. Mohammadi. Gharehghani: Analyzed and interpreted the data; Contributed reagents, materials, analysis tools, or data; Wrote the paper.

F Ebadi Fard Azar: Analyzed and interpreted the data.

G. Soofizad: Analyzed and interpreted the data; Wrote the paper.

### Funding statement

This work was supported by 10.13039/501100005317Kermanshah University of Medical Sciences.

### Data availability statement

Data will be made available on request.

### Declaration of interests statement

The authors declare no conflict of interest.

### Additional information

No additional information is available for this paper.
